# Tissue, cellular, and molecular level determinants for eye lens stiffness and elasticity

**DOI:** 10.3389/fopht.2024.1456474

**Published:** 2024-08-08

**Authors:** Catherine Cheng

**Affiliations:** School of Optometry and Vision Science Program, Indiana University, Bloomington, IN, United States

**Keywords:** actin, Eph, ephrin, CP49, interdigitations, capsule, suture

## Abstract

The eye lens is a transparent, ellipsoid tissue in the anterior chamber that is required for the fine focusing of light onto the retina to transmit a clear image. The focusing function of the lens is tied to tissue transparency, refractive index, and biomechanical properties. The stiffness and elasticity or resilience of the human lens allows for shape changes during accommodation to focus light from objects near and far. It has long been hypothesized that changes in lens biomechanical properties with age lead to the loss of accommodative ability and the need for reading glasses with age. However, the cellular and molecular mechanisms that influence lens biomechanical properties and/or change with age remain unclear. Studies of lens stiffness and resilience in mouse models with genetic defects or at advanced age inform us of the cytoskeletal, structural, and morphometric parameters that are important for biomechanical stability. In this review, we will explore whether: 1) tissue level changes, including the capsule, lens volume, and nucleus volume, 2) cellular level alterations, including cell packing, suture organization, and complex membrane interdigitations, and 3) molecular scale modifications, including the F-actin and intermediate filament networks, protein modifications, lipids in the cell membrane, and hydrostatic pressure, influence overall lens biomechanical properties.

## Introduction

The function of the eye lens to fine-focus light onto the retina depends on tissue transparency, high refractive index, and biomechanical integrity and resilience. In humans, the lens changes shape during a process called accommodation to focus light coming from different distances to transmit a clear image. Accommodation depends on the biomechanical properties of the lens, ciliary muscles, and zonular fibers. Previous work has studied several factors that may be involved in the age-related loss of accommodative amplitude, including ciliary muscle weakening, zonular fiber changes in elasticity, and increased lens stiffness. Studies have shown no age-related loss of zonular fiber elasticity (1, 2), and while there are age-related changes in the ciliary muscles and its connection to the peripheral tissues of the eye (1, 3, 4), the major contributing factor for presbyopia appears to be lens biomechanical properties (5). Age-related changes in lens stiffness and elasticity have been linked to the loss of accommodative amplitude leading to presbyopia and the need for reading glasses (6–17). There are many theories regarding how lens stiffness increases with age, but the cellular and molecular mechanisms that drive these biomechanical changes are just beginning to be understood.

**Figure 1:**
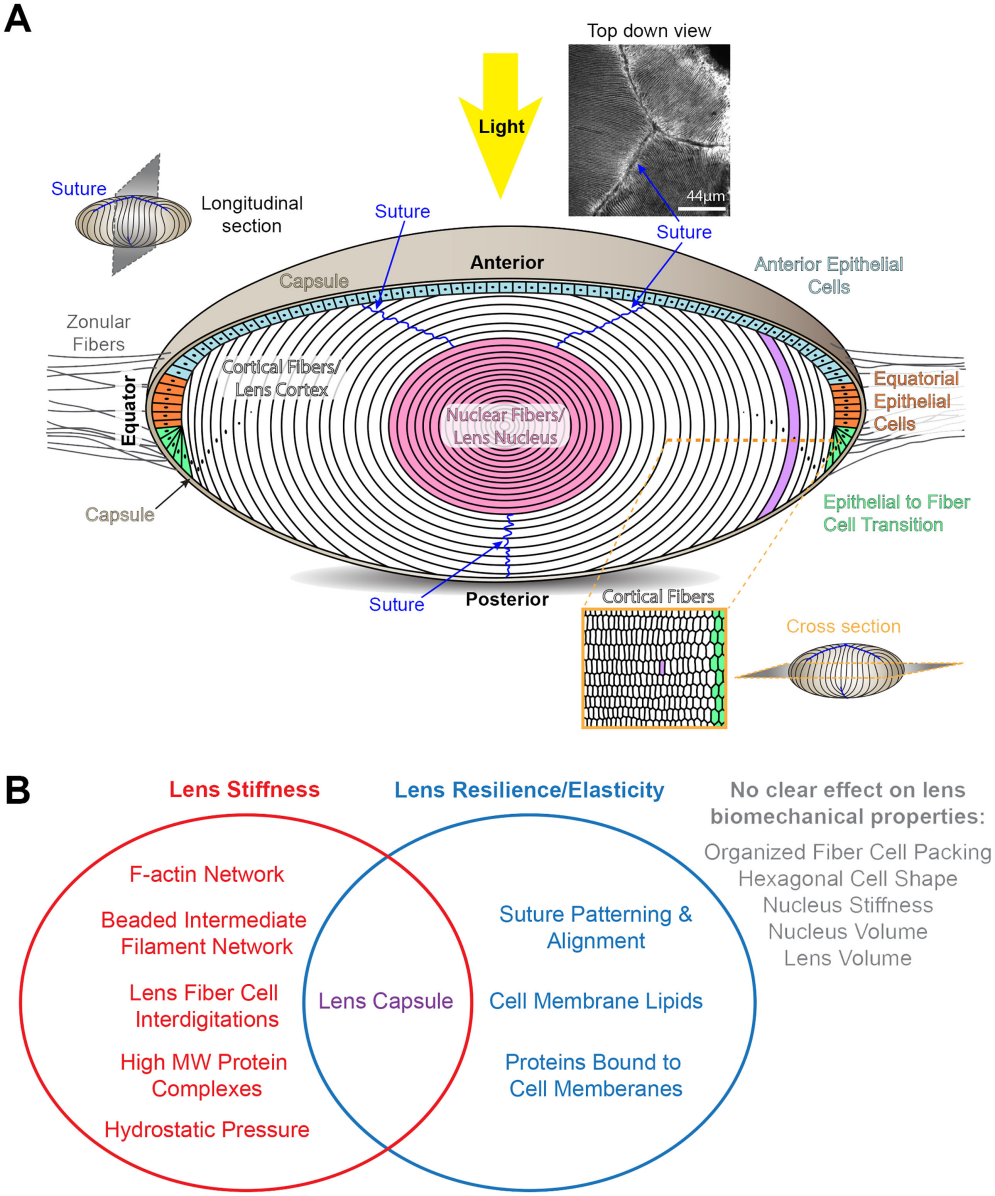
Diagrams of the structure of the lens and factors that influence lens biomechanical properties. **(A)** The cartoon of the lens shows a longitudinal plane. The lens is surrounded by a basement membrane called the capsule (tan). The lens is suspended in the anterior chamber of the eye by zonular fibers (gray lines) extending from the ciliary body that are anchored into the lens capsule. Light rays enter the lens from the anterior pole. Lens epithelial cells form a monolayer that covers the anterior hemisphere of the lens. The anterior epithelial cells (light blue) are quiescent. Equatorial epithelial cells (orange) proliferate, and one of the daughter cells (green) will start to differentiate into lens fibers cells. The bulk of the lens is made up of fiber cells (white and pink). These long and skinny cells are hexagonal in cross section (lower right cartoon). Newly formed fiber cells will elongate toward the anterior and posterior poles, and the tips of these fiber cells will form the suture (blue lines). A photo of the lens suture stained for F-actin to mark the cell boundaries is shown on the upper right. During fiber cell maturation, there is a process that removes all cellular organelles to prevent light scattering. Fiber cells are highly compacted at the center of the lens, also known as the lens nucleus (pink). Scale bar for suture picture, 44μm. The rest of the cartoons are not drawn to scale. **(B)** A Venn diagram summary of the tissue, cellular, and molecular factors that influence lens stiffness and/or resilience.

The lens is surrounded by a thin collagenous basement membrane, known as the capsule, and is composed of two cell types, epithelial and fiber cells (**Figure 1A**). A monolayer of lens epithelial cells covers the anterior hemisphere of the tissue, and most of the lens consists of a large mass of fiber cells (18). Anterior quiescent epithelial cells are cuboidal in shape and cobblestone in cross section. These cells do not normally proliferate and are thought to help maintain the adjacent lens fiber cells. Epithelial cells at the lens equator in the germinative zone proliferate, migrate, and differentiate into fiber cells. The lifelong growth of the lens depends on the continuous and concentric addition of new fiber cell shells (19, 20). The oldest cells of the lens are in the inner portions of the tissue, and no cells are shed from the lens due to the lens capsule. Newly formed fiber cells elongate toward the anterior pole with their apical tips crawling along the apical surface of anterior epithelial cells while their basal tips migrate toward the posterior pole along the posterior lens capsule. When elongating fiber cell tips reach the anterior or posterior pole, the tips detach from the anterior epithelial cells or posterior capsule and contact fiber cell tips that extend from opposing sides of the tissue to form the suture (21–23). The suture solves the geometric problem that the fiber cells tips can never narrow enough to fully reach the point of the anterior and posterior poles (20). Fiber cells are long and skinny cells that are hexagonal in cross section with complex interdigitations forming a 3-dimensional zipper along the cell length (18, 24–27). The patterning of the interdigitations changes during fiber cell maturation and are hypothesized to affect lens biomechanical properties (28, 29). Fiber cells are supported by F-actin and specialized beaded intermediate filament networks, composed of CP49 and filensin (30–32). As fiber cells continue to mature, the cells undergo a process to remove all cellular organelles (18, 33) and are compacted toward the center of the tissue, also known as the lens nucleus, into a region of increased stiffness (6, 14, 18, 24, 34–36).

Many methods have been used to determine stiffness and elasticity in human lenses (6, 10, 12–15, 37–40) and animal lenses (34, 41–55). Although animal lenses do not accommodate like human lenses, animal lenses increase in stiffness with age in a similar manner (34, 35, 41, 43–45, 52–54). In addition, mouse models offer the ability to study age-related changes in a relatively shortened period of time with the average lifespan of laboratory mice being 26-29 months (56, 57). Genetic models of knockout, knock-in, and transgenic mice allow the dissection of pathways required for lens homeostasis and biomechanics. It should be noted that murine lenses are nearly spherical in shape compared to human lenses that are ellipsoid in shape, and rodent lenses have much stiffer lens nuclei compared to primate lenses. Structurally, human and mouse lens fibers share similar morphologies, interdigitations, and patterning, but human lenses have more complex sutures (21, 58). While studies of mouse models can offer insights into lens tissue mechanical properties, murine lenses do not accommodate like human lenses.

In this review, we will discuss the various factors that have been hypothesized to affect lens biomechanics and highlight data from human and animal studies that reveal tissue, cellular, and molecular scale changes that influence lens stiffness and resilience or elasticity (**Figure 1B**).

## Tissue organization and integrity

### Lens capsule

The capsule surrounding the lens is mainly composed of collagen IV, laminin, and perlecan (59–71), and this basement membrane prevents the shedding of lens cells during development and aging (18). The capsule shapes the lens during accommodation (72). With age, the human lens capsule increases in thickness, stiffens, becoming more brittle (12, 73–77). Additionally, measurements of biomechanical properties of decapsulated human and animal lenses show that removal of the lens capsule causes changes in lens shape (46), a decrease in stiffness (40, 50, 73), and altered viscoelastic properties (46). Based on lens capsule and whole lens stretching studies, in samples from older humans and primates, the stiffness of the lens capsule alone does not determine overall tissue stiffness and, most likely, does not contribute to presbyopia (78). In mouse lenses, the capsule increases in thickness up to 4 months of age, and the continued age-related increase in lens stiffness is not linked to capsule thickness (35). Measurements of human lens stiffness with a spinning method showed larger post-spinning equatorial diameters in decapsulated lenses than intact lenses (40), suggesting that the lens capsule plays a role in resilience of the lens to recover its shape after deformation. From these data, while the lens capsule is important for overall tissue biomechanics and shape, this basement membrane is unlikely to be the major factor in the age-related stiffening of the lens.

### Lens volume, nucleus volume, and nucleus stiffness

It has been theorized that increased lens volume, nucleus volume, or nucleus stiffness with age lead to an overall increase in human lens stiffness with age, leading to presbyopia (6, 47, 79–81). In isolated fiber cells from the cortex or nucleus of sheep lenses, atomic force microscopy (AFM) showed that nuclear fibers were stiffer than cortical fibers (55). Our data in aging mice showed that the lens volume increases with age until the volume plateaus at 18 months of age (35). Lens stiffness in aged mice continues to increase up to 30 months of age, and thus, lens volume increases are unlikely to affect lens stiffness in old mice (35). In aged mice, the volume of the lens nucleus increases with age, and there is a large jump in lens nucleus volume between 24 months and 30 months of age without a similarly large jump in lens stiffness at 30 months of age (35). Genetic knockout or knockdown mouse models can lead to opposing changes in lens nucleus volume. Loss of EphA2, a receptor tyrosine kinase, results in a smaller and softer lens nucleus without any changes in overall lens stiffness (82, 83). In contrast, knockdown of tropomyosin 3.5 (Tpm3.5), an F-actin stabilizing protein, resulted in a larger and stiff lens nucleus, but these knockdown lenses were soft at high compressive loads (84). From these data, lens volume, nucleus volume, and nucleus stiffness are unlikely to play significant roles in overall lens stiffness, at least in rodent lenses. In human lenses, an alternate theory proposes that the restriction of cytoplasmic movement in the lens nuclear fibers can lead to increased lens stiffness and decreased accommodation with age (85). This idea is discussed below in the section on lens protein modifications with age.

The relationship between lens resilience or elasticity with lens and nucleus volumes is unclear. Lens resilience, measured by axial diameter recovery in 2- to 24-month-old mice after compression, was ~94-96% of the pre-compression axial diameter (35). Interestingly, in very old mice, lens resilience is nearly 99% (35). This jump in lens resilience is not correlated with lens volume since the lens stops increasing in volume by 18 months of age (35). The increased lens resilience in 30-month-old mice could be related to the significant increase in nucleus volume (35). Alternatively, the lenses from 30-month-old mice are very stiff and do not compress much; thus, these lenses recover more fully after load removal.

## Cellular arrangement and packing

### Hexagon cell shape and organized fiber cell packing

The correlation between hexagon cell shape and the organized packing of lens fiber cells on lens stiffness is weak. Studies of mouse models with a disturbance of cell membrane-associated proteins, periaxin and ankyrin-B, showed changes in fiber cell shape and decreased lens stiffness (86). These abnormal fibers appear polygonal but differ in width and height compared to the uniform cell size in control lens fibers (86–88). Further work revealed that changes in fiber cell hexagon cell shape and disorganization of fiber cell packing does not affect lens stiffness. Loss of tropomodulin 1 (Tmod1), an actin pointed-end capping protein, in the lens only leads to a mild change in lens stiffness at low compressive loads (41, 89). The loss of EphA2 or mutations in myosin IIA (NMIIA) lead to obvious defects in fiber cell packing and hexagon cell shape, but no changes in lens stiffness (82, 90–92). Further, in aged mice, there is a loss of hexagon cell shape, uniform fiber cell size, and organized fiber cell packing, but lenses from the very old mice are very stiff. Thus, the collective evidence suggests that organized fiber cell packing and hexagon cell shape does not affect lens stiffness. It is likely that decreased levels of cytoskeletal proteins in homozygous periaxin knockout and heterozygous ankyrin-B knockout lenses lead to changes in stiffness rather than just simply a change in cell shape, as discussed below.

In the mutant mouse models described above with disordered lens fibers, resilience is increased in very old mice and in EphA2 knockout lenses. The increased lens resilience in very old mice happens suddenly at 30 months of age, but the hexagonal cell shape changes are apparent by 12 months of age. Thus, in this case, it is unlikely that ordered fiber cell packing affects lens resilience. For the EphA2 knockout lenses, the reason for increased resilience is due to the lens suture changes discussed in detail below.

### Lens suture

The lens suture structure differs between non-accommodating and accommodating species. Typically, in non-accommodating mouse lenses, there are well-aligned Y-shape sutures with 3 branches, and there are a low percentage of wild-type lenses that exhibit sutures with additional branches (42, 82). Each layer of the Y-suture in mouse lenses is overlaid precisely on previously shells of lens fiber cells (42, 82). In contrast, in accommodating species, lens sutures begin as a 3-pronged Y shape and continue to branch with the addition of new layers of lens fibers (21, 58). Studies of the EphA2 and ephrin-A5 knockout mice have revealed the role of the Y-suture in lens resilience. Ephrin-A5 is part of a family of ligand proteins that bind to Eph receptors. Loss of either EphA2 or ephrin-A5 result in misaligned shells of lens fiber cells that have abnormal lens suture branching (82, 93). There are no changes in lens stiffness in either EphA2 or ephrin-A5 knockout lenses, but there is increased lens resilience, and knockout lenses recovered more completely after load removal (82). This change in lens resilience is not related to disorganized fiber cell packing because the ephrin-A5 knockout lenses have increased resilience with normal, organized, and hexagonal lens fiber cells (94, 95). Thus, this data indicates that Y-shaped sutures in mouse lenses constrain the resilience of the tissue. It should be noted that these biomechanical studies compressed the lenses with a high load that resulted in higher than physiological strains on the lens. When control and knockout lenses were compressed to physiological strains, both groups of samples recovered completely after load removal.

### Lens fiber cell interdigitations

It has long been hypothesized that the complex interdigitations that create a 3D zipper between fibers cells is required for lens biomechanical properties (28, 29). This elegant pattern of interlocking membrane structures changes during fiber cell differentiation and maturation, and has been characterized extensively by electron microscopy (24–29, 35, 83, 84, 96–107). During lens fiber cell maturation, the loss of cellular organelles is accompanied by the appearance of large interlocking paddle domains between neighboring cells (83, 84, 96–98). In Tmod1 knockout lenses, which are softer at low mechanical loads (41), the large paddle domains do not form between these knockout fiber cells (96). Tmod1 interacts with the spectrin-actin network and is normally associated with β2-spectrin at the fiber cell membrane in the valleys between large paddle domains. Loss of Tmod1 causes dissociation of β2-spectrin from the cell membrane and abnormal distribution of α-actinin, a crosslinking protein for antiparallel actin filaments, along the cell membrane (89). This is the first direct evidence that fiber cell interdigitations influence lens stiffness. There is no change in lens resilience in these knockout lenses, and it remains unknown whether fiber cells interdigitations play a significant role in lens elasticity.

## Molecular level alterations

### The F-actin network and associated proteins

The F-actin network plays a crucial role in lens fiber cell shape and patterning [reviewed in (30)]. When Tpm3.5 is knocked down in mouse lenses, there is a significant decrease in stiffness at high compressive loads (84). Electron microscopy and single fiber cell staining studies reveal no obvious changes in fiber cell interdigitations, but the decrease in Tpm3.5 at the fiber cell membrane causes a reorganization of the F-actin network. While the protein levels remained unchanged, there was an expansion of the β2-spectrin- and α-actinin-associated F-actin networks at the cell membrane displacing the fimbrin, also known as plastin, bundled F-actin network. The alteration in the type of F-actin network at the fiber cell membrane is correlated with decreased stiffness of the knockdown lens.

Several studies have looked at the effects of actin polymerization, myosin motor domain, and myosin light chain kinase inhibition on lens stiffness. Treatment of chick lenses *ex vivo* with F-actin and myosin disruptors, including latrunculin, blebbistatin, and ML-7, resulted in softer lenses under whole tissue compression tests, and the effects of treatments were reversible (108). The stiffness changes were accompanied by a decrease in the levels of F-actin and phosphorylated myosin along with dissociation of F-actin and myosin from the basal surface of fiber cells. Presumably, shorter actin filaments or disruption of myosin motor activities leads to the softening of treated lenses. In contrast, AFM studies of sheep lens fibers treated with cytochalasin D, an inhibitor of F-actin polymerization, or nocodazole, an inhibitor of microtubule polymerization, showed no changes in local stiffness of the fiber cells (55). Since the lenses were flash frozen, stored at -80°C, and thawed before cell dissociation with chelating agents, the cells were presumably no longer metabolically active. Thus, the use of cytoskeletal inhibitors in this study may have little effect on these thawed cells.

Interestingly, recent studies of transgenic mice with human mutations in NMIIA showed no change in lens stiffness (91, 92). One mutation was in the motor domain of NMIIA, and two mutations were in the tail domain. It should be noted that the motor domain mutation and one of the tail domain mutations are homozygous embryonic lethal, and therefore, in the heterozygous transgenic mice, compensation by the endogenous wild-type protein may affect the biomechanical testing results.

Reports of softer lenses due to loss of periaxin or decreased levels of ankyrin-B are accompanied by decreased protein levels of β-actin, β2-spectrin, desmoyokin, NrCam, activated myosin, and calcium channels as well as changes in the localization of membrane cytoskeleton proteins in immunostained lens fibers (86, 87). The changes in fiber cell shape and width are likely downstream of the loss of these membrane proteins that are required for normal cell architecture. These data support the notion that lens stiffness depends on a normal F-actin network.

The role of the F-actin network in lens resilience is unclear. While loss of Tmod1 slightly alters lens stiffness at low compressive strains, the loss of Tmod1 and mutations in NMIIA do not affect lens resilience (41, 91, 92). The Tpm3.5 knockdown lenses have decreased lens resilience, but this is likely due to high strains on the softer knockdown lenses that are unable to recover fully after load removal.

### Beaded intermediate filaments

Lens fiber cells are supported by a specialized beaded intermediate filament network, composed of two proteins, CP49 (also known as phakinin) and filensin [reviewed in (32)]. Initially, the “beaded” structure of these intermediate filaments was erroneously attributed to the binding of α-crystallin proteins (109, 110). There is a spontaneous mutation in CP49 in the 129 mouse strain that leads to loss of CP49 (111, 112). Deletion of either CP49 or filensin causes complete loss of the beaded intermediate filaments in the lens (112–115). CP49 knockout lenses are softer than control lenses (41, 43). The contribution of CP49 to lens diameter and formation of the lens nucleus is unclear. One report suggests loss of CP49 leads to decreased lens diameters and nucleus diameters (43) while another study shows no difference in lens volumes and nuclear volumes when compared to control lenses (41). Both studies showed that resilience of lenses without CP49 is comparable to control lenses (41, 43). Thus, the loss of beaded intermediate filaments leading to softer lenses without a change in lens elasticity is presumably due to weakening of the fiber cell cytoskeleton. The conflicting reports of lens and nucleus diameter and volume changes in mice with CP49 disruption are not clearly linked to the change in lens stiffness.

### Protein modifications and crosslinking

For transparency and high refractive index, lens cells contain an extraordinarily high amount of proteins (~450mg/ml), and 90% of all lens proteins are crystallins (18). Crystallins are divided into three families in humans, α, β, and γ. Since there is little or no protein turnover and no loss or renewal of cells in this tissue, the proteins made during initial embryonic development are present in the nuclear fiber cells at the center of the lens for an entire lifetime. There have been many studies showing post-translational modification, glycation, and aggregation of crystallins with age. Alpha-crystallins, members of the small heat shock protein family, have been shown to have chaperone-like activity and act as sinks to sequester misfolded or unfolded proteins to prevent protein aggregation (116, 117). The other major classes of crystallins belong to the family of β/γ crystallins that function as structural proteins that bind calcium in the lens (116, 118–121). By 35-45 years of age in human lenses, there is significant loss of α-crystallin proteins from the soluble protein fractions in the lens nucleus, and at the same time, there is an increase of insoluble high molecular protein species (7, 122, 123). This change in protein solubility coincides with the typical onset age for presbyopia, and it is hypothesized that the increased size and stiffness of high molecular weight protein species may affect the ability for the lens nucleus to deform during accommodation (124), supporting the theory that the cytoplasm of aged nuclear fibers has restricted movement (85).

During aging, human and mouse lenses have increased levels of advanced glycation end products (AGEs) (125–133). Studies of mouse lenses after thermal stress revealed changes in lens stiffness that were correlated with increased AGEs (132, 134). Treatment of mouse lenses under thermal stress with a plant flavanone, hesperetin, prevents the increase in AGEs and the increase in lens stiffness. However, heating the tissue may cause other types of damage to lead to the change in lens stiffness. In addition to glycation, lysine acylation is a common type of protein modification. In the lens, changes in lysine have been detected as one of the major post-translational protein modifications (135–137). Human and mouse lenses treated *in vitro* and *in vivo* with aggrelyte-2 had reduced lens stiffness and increased water-soluble proteins, presumably through protein acetylation and breaking of disulfide bonds (138, 139). Many other post-translational modifications of crystallins have been described in aging lenses, including phosphorylation, deamindation, methylation, racemization, isomerization, lipidation, oxidation, and truncation [reviewed in (140–143)]. Studies have largely linked these protein changes to cataracts. Presumably, any protein modifications that results in disulfide bond formation or crosslinking can lead to changes in lens stiffness, but there have not yet been any direct experiments to address the possible changes in biomechanics due to specific post-translational protein modifications.

The strategy to break disulfide protein bonds to reverse presbyopia has previously been explored through the application of α-lipoic acid to animal and human lenses. There was encouraging data from old animal and *in vitro* human studies that this agent could soften lenses (144–146). Unfortunately, the formulation of α-lipoic acid UNR844 being developed by Novartis failed in phase 2b clinical trials, and this potential treatment for presbyopia has been abandoned (147).

### Lipids in the cell membrane

The lipid content of human lens fiber cell membranes changes with age until about 40-45 years of age (148, 149), coinciding with the onset of presbyopia. Cholesterol content influences cell membrane rigidity, and lens fiber cell membranes have unusually high levels of cholesterol (150–154). High cholesterol contents lead to the formation of cholesterol bilayer domains in lens fiber cell membranes (155). AFM studies suggest that cholesterol content in the fiber cell membranes regulates cell elasticity (156). Age-related changes in the cholesterol species in fiber cell membranes (150) and the binding of α-crystallins to the cell membrane with age (157–163) are thought to lower lens deformability with age (164).

### Hydrostatic pressure and osmotic balance

Hydrostatic pressure is maintained in the lens by a network of sodium channels, gap junctions composed of connexins, and water channels composed aquaporins (165). There is a steady increase in hydrostatic pressure in the lens with age (166). Loss of connexin 46 (Cx46 or α3) leads to stiffening of knockout lenses possibly due to protein degradation and nuclear cataracts (48). It is worth noting that heterozygous Cx46 knockout lenses have elevated hydrostatic pressure (165), and therefore, it is also possible that the loss of Cx46 causes increased lens stiffness due to the disruption of the gap junctions’ ability to regulate lens hydrostatic pressure. Water channels, made of aquaporin 0, are abundant in the lens fiber cells and are thought to facilitate intercellular water transport in the lens cortex (167–169). Aqp0 is known to be cleaved during fiber cell differentiation leading to a loss of water channel function, and cleaved Aqp0 is thought to function as cell-cell adhesion molecules in mature fiber cells (170–173). Heterozygous and homozygous Aqp0 knockout lenses demonstrate changes in lens fiber cell shape, enlarged extracellular spaces, and these mutant lenses are softer than control lenses (45). Thus, the loss of Aqp0 in lenses can lead to changes in lens stiffness due to altered hydrostatic pressure and changes in fiber cell structure and stability (45, 174).

The hydrostatic pressure in the lens may be influenced by the ratio of bound vs. free water in the lens. Water is bound to the abundant amount of crystallin proteins in the lens, and during pressure changes, which can occur during accommodation, bound water is released from proteins resulting in an increase of free water in the lens, and thus decreasing the osmotic pressure (175–178). This process is known as syneresis. The ratio of free-to-bound water decreases with age leading to increased bound water and increased internal hydrostatic pressure as accommodative ability decreases (179–181). The change in free water content in the aging lens has been proposed as a possible contributor to lens stiffening with age and cataract formation (182).

## Concluding thoughts

Studies of animal and human lenses have revealed that lens stiffness is influenced by the capsule, complex interdigitations between fiber cells, the F-actin and beaded intermediate filament networks, age-related protein modifications, hydrostatic pressure, and osmotic balance. Lens elasticity is linked to the capsule, suture alignment, and the lipid and protein composition of fiber cell membranes. Surprisingly, animal lens studies do not show a clear link between lens volume, nucleus volume, nucleus stiffness, or organized fiber cell packing with overall lens biomechanical properties (**Figure 1B**). A common factor in regulating lens stiffness and elasticity is the integrity of the capsule. This important basement membrane is required to hold all lens cells together and provides the anchoring point for zonular fibers. While the contribution of the capsule to the development of presbyopia remains unclear, the contribution of various extracellular matrix components of the lens capsule to tissue biomechanics remains to be studied.

Lens fiber cell interdigitations are affected by cytoskeletal protein complexes, and both factors are required for normal lens stiffness. The mechanism for how fiber cell morphology is modulated by cytoskeletal proteins is unknown, and the signaling pathways required for normal fiber cell interdigitation development are still being studied. Protein modifications affect protein-protein interactions that in turn modulate osmotic balance and hydrostatic pressure. Changes in this balance lead to altered lens stiffness, and protein-protein disulfide bonds are now a target for pharmaceutical intervention for preventing, delaying, or even reversing presbyopia.

The patterning of the lens suture affects tissue elasticity likely though a change in the distribution of forces in lenses with branched sutures. These data support the known differences between accommodating and non-accommodating species where accommodating and more elastic lenses have high branched sutures. The age-related change in composition of the lipids and proteins bound to the fiber cell membrane suggests that the fluidity of cell membranes also influences lens resilience. The interconnected nature of tissue, cellular, and molecular level alterations that affect lens biomechanical properties with age suggest that strategies to prevent or reverse presbyopia may need to target multiple factors that influence the stiffening of the lens.

## Author contributions

CC: Conceptualization, Funding acquisition, Project administration, Writing – original draft, Writing – review & editing.
